# Comparison of early and late *Pneumocystis jirovecii* Pneumonia in kidney transplant patients: the Korean Organ Transplantation Registry (KOTRY) Study

**DOI:** 10.1038/s41598-022-14580-5

**Published:** 2022-06-23

**Authors:** Gongmyung Lee, Tai Yeon Koo, Hyung Woo Kim, Dong Ryeol Lee, Dong Won Lee, Jieun Oh, Beom Seok Kim, Myoung Soo Kim, Jaeseok Yang, KOTRY Study Group, Myoung Soo Kim, Jaeseok Yang, Jin Min Kong, Oh Jung Kwon, Deok Gie Kim, Cheol Woong Jung, Yeong Hoon Kim, Joong Kyung Kim, Chan-Duck Kim, Ji Won Min, Sik Lee, Yeon Ho Park, Jae Berm Park, Jung Hwan Park, Jong-Won Park, Tae Hyun Ban, Sang Heon Song, Seung Hwan Song, Ho Sik Shin, Chul Woo Yang, Hye Eun Yoon, Kang Wook Lee, Dong Ryeol Lee, Dong Won Lee, Jieun Oh, Sang-Ho Lee, Su Hyung Lee, Yu Ho Lee, Jung Pyo Lee, Jeong-Hoon Lee, Jin Seok Jeon, Heungman Jun, Kyung Hwan Jeong, Ku Yong Chung, Jong Soo Lee, Ju Man Ki, Dong-Wan Chae, Soo Jin Na Choi, Sung Shin, Seungyeup Han, Kyu Ha Huh

**Affiliations:** 1 Division of Nephrology, Department of Internal Medicine, Yonsei University College of Medicine, Severance Hospital, 50 Yonsei-ro, Seodaemun-gu, Seoul, 03722 Republic of Korea; 2 Division of Nephrology, Department of Internal Medicine, Korea University Anam Hospital, Seoul, Republic of Korea; 3 Department of Internal Medicine, Maryknoll Medical Center, Pusan, Republic of Korea; 4 Department of Internal Medicine, Pusan National University School of Medicine, Pusan, Republic of Korea; 5 Department of Internal Medicine, Kangdong Sacred Heart Hospital, Hallym University College of Medicine, Seoul, Republic of Korea; 6 Department of Surgery, Gangnam Severance Hospital, Yonsei University College of Medicine, Seoul, Republic of Korea; 7 Department of Nephrology, BHS Hanseo Hospital, Pusan, Republic of Korea; 8 Department of Surgery, College of Medicine, Han Yang University, Seoul, Republic of Korea; 9 Department of Surgery, Yonsei University Wonju College of Medicine, Wonju Severance Christian Hospital, Wonju, Republic of Korea; 10 Department of Surgery, Korea University Anam Hospital, Seoul, Republic of Korea; 11 Department of Internal Medicine, Inje University Busan Paik Hospital, Pusan, Republic of Korea; 12 Department of Internal Medicine, Bongseng Memorial Hospital, Pusan, Republic of Korea; 13 Department of Internal Medicine, School of Medicine, Kyungpook National University Hospital, Daegu, Republic of Korea; 14 Division of Nephrology, Department of Internal Medicine, Bucheon St. Mary’s Hospital, Bucheon, Republic of Korea; 15 Department of Internal Medicine, Jeonbuk National University Hospital, Jeonju, Republic of Korea; 16 Department of Surgery, Gil Medical Center, Gachon University College of Medicine, Incheon, Republic of Korea; 17 Department of Surgery, Samsung Medical Center, Sungkyunkwan University School of Medicine, Suwon, Republic of Korea; 18 Department of Nephrology, Konkuk University School of Medicine, Seoul, Republic of Korea; 19 Department of Nephrology, Yeungnam University Hospital, Daegu, Republic of Korea; 20 Division of Nephrology, Department of Internal Medicine, Eunpyeong St. Mary’s Hospital, Seoul, Republic of Korea; 21 Department of Internal Medicine, Pusan National University Hospital, Pusan, Republic of Korea; 22 Department of Surgery, Ewha Womans University Seoul Hospital, Seoul, Republic of Korea; 23 Department of Internal Medicine, Division of Nephrology, Kosin University College of Medicine, Pusan, Republic of Korea; 24 Division of Nephrology, Department of Internal Medicine, Seoul St. Mary’s Hospital, Seoul, Republic of Korea; 25 Department of Internal Medicine, College of Medicine, Incheon St. Mary’s Hospital, The Catholic University of Korea College of Medicine, Bucheon, Republic of Korea; 26 Department of Nephrology, Chungnam National University Hospital, Daejeon, Republic of Korea; 27 Department of Nephrology, Kyung Hee University Hospital at Gangdong, Seoul, Republic of Korea; 28 Department of Surgery, Ajou University School of Medicine, Suwon, Republic of Korea; 29 Division of Nephrology, Department of Internal Medicine, CHA Bundang Medical Center, CHA University, Seongnam, Korea; 30 Department of Nephrology, SMG-SNU Boramae Medical Center, Seoul, Republic of Korea; 31 Department of Surgery, Myongji Hospital, Goyang, Republic of Korea; 32 Department of Internal Medicine, Soonchunhyang University Seoul Hospital, Seoul, Republic of Korea; 33 Department of Surgery, Inje University Ilsan Paik Hospital, Goyang, Republic of Korea; 34 Department of Internal Medicine, Kyung Hee University College of Medicine, Seoul, Republic of Korea; 35 Department of Surgery, Ewha Womans University Mokdong Hospital, Seoul, Republic of Korea; 36 Department of Surgery, Ulsan University Hospital, Ulsan, Republic of Korea; 37 Division of Nephrology, Seoul National University Bundang Hospital, Seoul, Republic of Korea; 38 Department of Surgery, Chonnam National University Medical School, Gwangju, Republic of Korea; 39 Department of Surgery, Asan Medical Center, Seoul, Republic of Korea; 40 Department of Internal Medicine, Keimyung University School of Medicine, Daegu, Republic of Korea

**Keywords:** Infectious diseases, Organ transplantation

## Abstract

Late *Pneumocystis jirovecii* pneumonia (PJP) is not rare in the era of universal prophylaxis after kidney transplantation. We aimed to determine the nationwide status of PJP prophylaxis in Korea and compare the incidence, risk factors, and outcomes of early and late PJP using data from the Korean Organ Transplantation Registry (KOTRY), a nationwide Korean transplant cohort. We conducted a retrospective analysis using data of 4,839 kidney transplant patients from KOTRY between 2014 and 2018, excluding patients who received multi-organ transplantation or were under 18 years old**.** Cox regression analysis was performed to determine risk factors for early and late PJP. A total of 50 patients developed PJP. The number of patients who developed PJP was same between onset before 6 months and onsets after 6 months. There were no differences in the rate, duration, or dose of PJP prophylaxis between early and late PJP. Desensitization, higher tacrolimus dose at discharge, and acute rejection were associated with early PJP. In late PJP, old age as well as acute rejection were significant risk factors. In conclusion late PJP is as common and risky as early PJP and requires individualized risk-based prophylaxis, such as prolonged prophylaxis for old patients with a history of rejection.

## Introduction

Pneumocystis jirovecii pneumonia (PJP) is a life-threatening opportunistic infection associated with increased mortality in kidney transplantation patients^1,2^. Before the era of prophylaxis, the incidence of PJP was reported as 0.6%–14%^2^ in kidney transplantation patients and was highest (6.5%–43%) in lung transplantation among solid organ transplantations^3^. Although the incidence of PJP has decreased in the era of universal prophylaxis, it is still as high as 0.4%–2.2%^4^ in kidney transplant patients. The introduction of potent immunosuppressants, such as tacrolimus, and pre-transplant desensitization for human leukocyte antigen (HLA)-incompatible or ABO-incompatible kidney transplantation, induction therapies, such as anti-thymocyte globulin (ATG), might have contributed to the persistently high prevalence of PJP in transplant patients despite PJP prophylaxis^5^.

PJP frequently occurs within 6 months after kidney transplantation, which is a critically immunocompromised period^6,7^. Cumulative doses of tacrolimus, mycophenolate mofetil, corticosteroids, ATG use, history of acute rejection, number of anti-rejection treatments, cytomegalovirus (CMV) infection, bacterial pneumonia, tuberculosis, and hepatitis C virus infection have been reported as risk factors for PJP^8–10^. Based on these epidemiologic data, the Kidney Disease Improving Global Outcomes (KDIGO) guidelines recommend universal PJP prophylaxis with daily trimethoprim–sulfamethoxazole (TMP-SMX) for the first 3–6 months after kidney transplantation, the American Society of Transplantation recommends prophylaxis for 6–12 months, and the European Renal Association recommends 12 months of prophylaxis when calcineurin inhibitors are given^11–13^. However, the dosage was not determined by a randomized control study, and no unified guidelines for the duration of prophylaxis have yet been established.

Recently, late-onset PJP 6 months after kidney transplantation has been reported in kidney transplant patients^14,15^. However, the risk factors and clinical outcomes of late-onset PJP compared to early-onset PJP have not been analyzed based on nationwide data. In this study, we aimed to determine the nationwide status of PJP prophylaxis in Korea and compare the incidence, risk factors, and outcomes of early and late PJP in kidney transplant patients using data from the Korean Organ Transplantation Registry (KOTRY), a nationwide Korean transplant cohort^16^. Thirty-two centers among 66 centers have voluntarily participated in the kidney transplant cohort in the KOTRY since 2014 and 82.8% of kidney transplant cases in Korea were enrolled.

## Results

### Prophylaxis regimens for PJP in Korea

**Figure 1:**
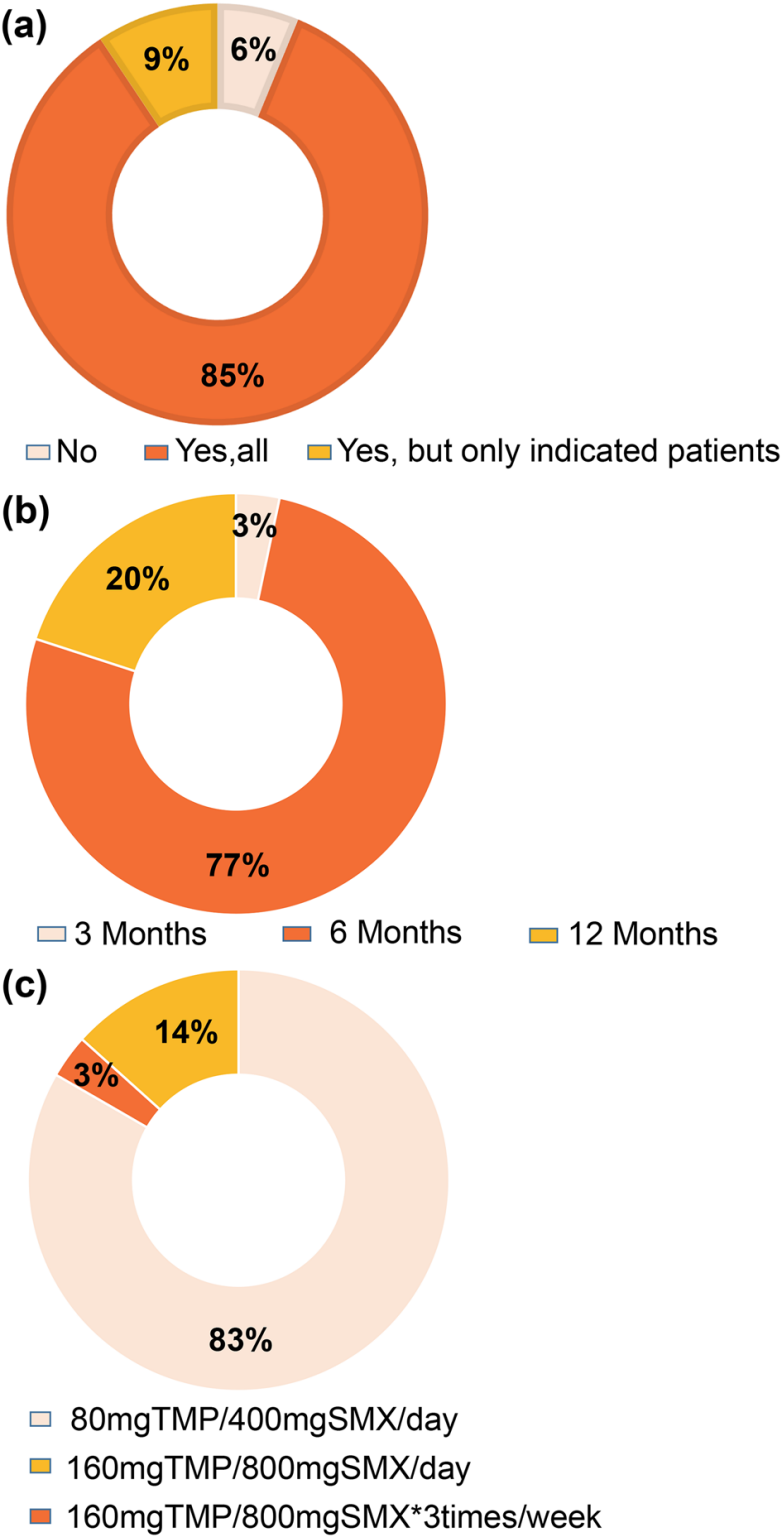
Prophylaxis regimens for *Pneumocystis jirovecii* pneumonia in Korea. (**a**) PJP prophylaxis policy of 32 transplantation centers. Indicated prophylaxis was applied to patients that had received desensitization, ATG or rituximab, or received anti-rejection therapy. (**b**) Duration of PJP prophylaxis. (**c**) Dose of for PJP prophylaxis. ATG, anti-thymocyte globulin; PJP, *Pneumocystis jirovecii* pneumonia; SMP, sulfamexothazole; TMP, trimethoprim.

PJP prophylaxis protocols were surveyed at all 32 transplantation centers participating in KOTRY and all centers responded to the survey. Policy of universal PJP prophylaxis to all recipients was adopted by 85% of transplantation centers and 9% of centers adopted indicated PJP prophylaxis for high-risk groups that received desensitization, ATG, rituximab, or anti-rejection treatment (Fig. 1a). However, 6% of the patients did not receive any PJP prophylaxis. The duration of PJP prophylaxis was 3, 6, and 12 months in 3%, 77%, and 20% of the centers, respectively (Fig. 1b). All centers used trimethoprim-sulfamethoxazole (TMP-SMX) as a prophylactic drug at different doses, such as daily single-strength dose (83%), double strength-dose thrice a week (3%), or daily double-strength dose (14%) (Fig. 1c). Secondary prophylactic drugs in cases of sulfa allergy were not covered in this survey.

### Baseline clinical characteristics of study population according to PJP

**Figure 2:**
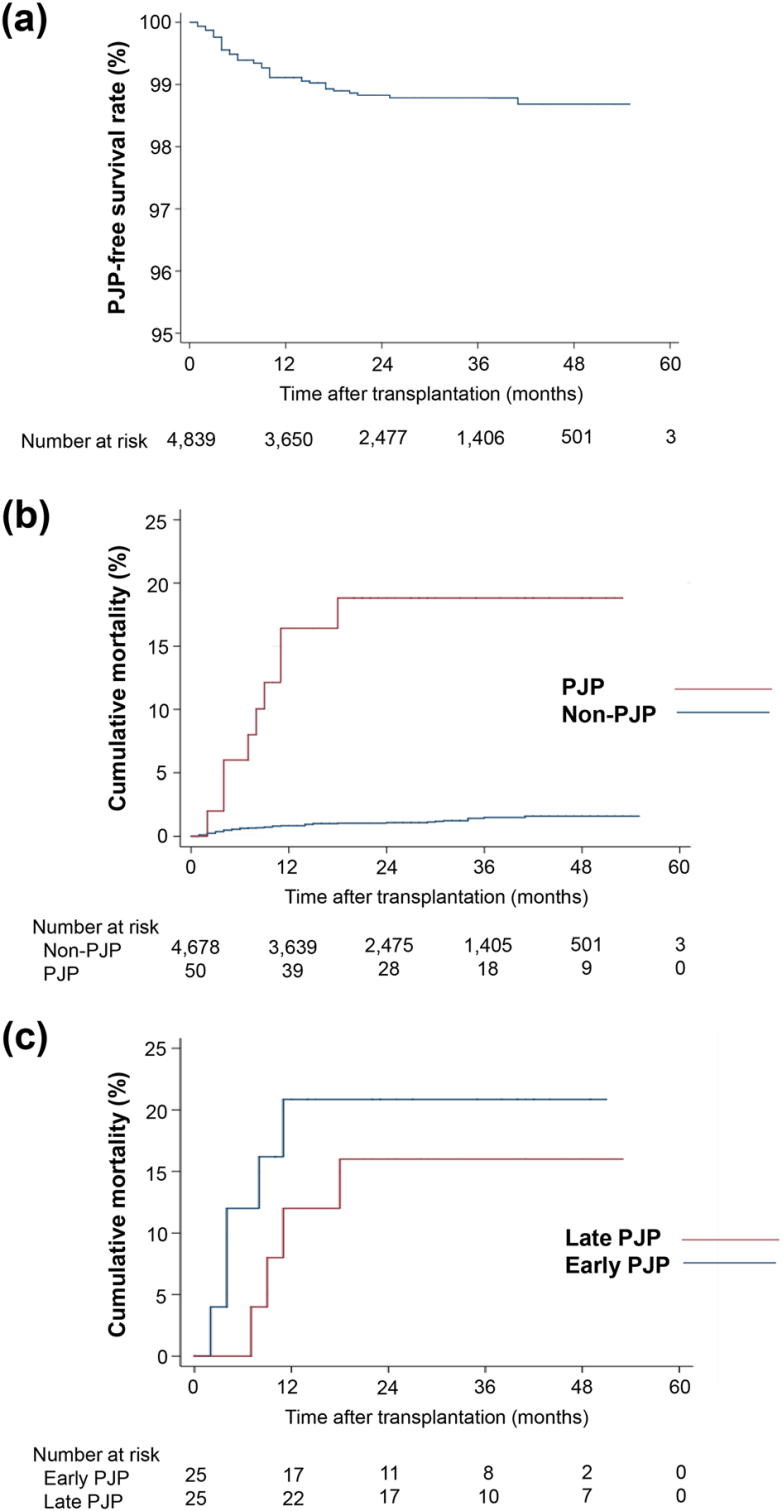
Development of *Pneumocystis jirovecii* pneumonia and its impact on mortality. (**a**) PJP-free survival rate after kidney transplantation. (**b**) Comparison of mortality between PJP and non-PJP groups (log rank test, *P* < 0.001). (**c**) Comparison of mortality between early and late PJP groups (log rank test, *P* = 0.546). PJP, *Pneumocystis jirovecii* pneumonia.

This study retrospectively analyzed 4,839 kidney transplant patients from 32 transplant centers in the KOTRY database between 2014 and 2018. Fifty of the 4,839 patients developed PJP after kidney transplantation, with an incidence of 4.90 per 1,000 patient-years (Fig. 2a). The baseline clinical characteristics of the study population were compared between patients with PJP and those without PJP (Table 1). The average age at transplantation was similar (49.1 ± 11.5 in the non-PJP group vs. 51.1 ± 13.6 in the PJP group, *P* = 0.102), and sex composition was also similar between the two groups. Desensitization was more commonly used, and the tacrolimus dose at discharge was higher in the PJP group than in the non-PJP group.

**Table 1:** Baseline characteristics of kidney transplant patients according to *Pneumocystis jirovecii* pneumonia.

Variables	Non-PJP (n = 4,789)	PJP (n = 50)	*P* value
Age at transplantation (years)	49.1 ± 11.5	51.1 ± 13.6	0.102
Gender (female)	1,945 (40.6%)	20 (40.0%)	0.930
**Smoking**			0.180
Never	3,633 (75.9%)	37 (74.0%)	
Current	413 (8.6%)	1 (2.0%)	
Former	691 (14.4%)	11 (22.0%)	
DM	1,428 (29.8%)	14 (28.0%)	0.780
BMI (kg/m^2^)	23.1 ± 0.10	21.80 ± 0.48	0.004
Hemoglobin (g/dL)	10.79 ± 2.74	10.63 ± 1.93	0.579
Serum creatinine (mg/dL)	1.29 ± 2.59	1.19 ± 0.39	0.169
eGFR (mL/min/1.73m^2^)	38.11 ± 36.12	35.60 ± 31.55	0.625
**Cause of ESRD**			0.282
DM, n (%)	1,125 (23.5%)	10 (20.0%)	
Hypertension, n (%)	757 (15.8%)	6 (12.0%)	
Glomerulonephritis, n (%)	1,588 (33.2%)	22 (44.0%)	
Others, n (%)	375 (7.8%)	6 (12.0%)	
Unknown, n (%)	944 (19.7%)	6 (12.0%)	
Delayed graft function	176 (3.6%)	4 (8.0%)	0.196
Re-transplantation, n (%)	372 (7.8%)	3 (6.0%)	0.450
**Type of donor**			0.289
Deceased donor, n (%)	3,004 (62.1%)	35 (70.0%)	
Living donor, n (%)	1,785 (36.9%)	15 (30.0%)	
Desensitization, n (%)	1,083 (22.5%)	19 (38.0%)	0.011
Number of HLA mismatch	3.2 ± 1.7	3.6 ± 1.4	0.104
Tacrolimus use at discharge, n (%)	4,608 (96.2%)	47 (94.0%)	0.433
Tacrolimus dose at discharge (mg)	5.96 ± 3.44	7.36 ± 4.62	0.014
Tacrolimus dose per body weight at discharge (mg/kg)	0.10 ± 0.00	0.13 ± 0.01	0.001
Tacrolimus level at discharge (ng/mL)	7.83 ± 0.05	8.05 ± 0.45	0.651
**Formulation of tacrolimus**			0.629
Once daily, n (%)	133 (2.7%)	0 (0.0%)	
Twice daily, n (%)	4,104 (85.69%)	37 (74%)	
Unknown, n (%)	371 (7.7%)	10 (20%)	
Tacrolimus conversion (twice to once daily), n (%)	156 (3.87%)	1 (3.03%)	1.000
ATG use, n (%)	998 (20.8%)	7 (14%)	0.236
Steroid use at discharge	4,690 (97.93%)	49 (98.0%)	*0.973*
MMF use at discharge	4,508 (94.13%)	46 (92.0%)	0.524
mTORi use at discharge	51 (1.06%)	2 (4.0%)	0.047
PJP prophylaxis, n (%)	4,626 (96.60%)	50 (100.0%)	0.184
**Dose of prophylactic TMP-SMP, n (%)**			0.038
Single strength/d or double strength × 3/wk	3,940 (82.27%)	48 (96.0%)	
Double strength/d	686 (14.32%)	2 (4.0%)	
Duration of PJP prophylaxis (months)	6.3 ± 2.6	6.0 ± 1.0	0.575
Mean follow up duration (months)	24.93 ± 15.32	8.42 ± 7.57	< 0.001

Data are presented as mean ± standard deviation, or n (%). *Abbreviations*: ATG, anti-thymocyte globulin; BMI, body mass index; d, days; DM, diabetes mellitus; eGFR, estimated glomerular filtration rate; ESRD, end stage renal disease; MMF, mycophenolate mofetil; mTORi, mammalian target of rapamycin inhibitor; N/A, not applicable; PJP, *pneumocystis jirovecii* pneumonia; TMP-SMX, trimethoprim/sulfamethoxazole; w, weeks.

PJP prophylaxis was administered to 4787 of 4839 patients. The duration of PJP prophylaxis was not significantly different between the two groups (6.3 ± 2.6 in the non-PJP group vs. 6.0 ± 1.0 in the PJP group, *P* = 0.575, Table 1). However, the daily double-strength dose of TMP-SMX was more commonly used in the non-PJP group (*P* = 0.038). Half of the PJP cases occurred within 6 months after transplantation, 15 (30.0%) occurred between 6 and 12 months, and 10 (20.0%) occurred after 12 months (Table 2). The mean time to PJP onset after termination of prophylaxis was 3.9 ± 6.6 months.

**Table 2:** Clinical characteristics and impacts of *Pneumocystis jirovecii* pneumonia on outcomes.

	Non-PJP (n = 4,789)	PJP (n = 50)	*P * value
**Onset after transplantation (months)**		8.4 ± 7.6	
0–6 months -n (%)		25 (50.0%)	
6–12 months -n (%)		15 (30.0%)	
12–24 months -n (%)		8 (16.0%)	
24–36 months -n (%)		1 (2.0%)	
36–48 months -n (%)		1 (2.0%)	
Graft rejection before PJP -n (%)	N/A	13 (26.0%)	
Time from rejection to PJP (months)	N/A	5.9 ± 3.6	
CMV infection before PJP -n (%)	N/A	1 (2.0%)	
Graft loss	103 (2.2%)	4 (8.0%)	0.024
Death	75 (1.6%)	9 (18.0%)	< 0.001
Death due to PJP	N/A	7 (14.0%)	

Note: data are presented as mean ± standard deviation, or n (%).

*Abbreviations*: CMV, cytomegalovirus; N/A, not applicable; PJP, *pneumocystis jirovecii* pneumonia.

### Impact of PJP on clinical outcomes after kidney transplantation

Kidney allograft rejection occurred in 523 of 4,839 patients. Thirteen cases of rejection occurred before PJP onset, and the average period from rejection to PJP occurrence was 5.9 ± 3.6 months. One case of CMV infection occurred before PJP. Graft failure occurred in 103 cases (2.2%) in the non-PJP group and in four cases (8.0%) in the PJP group (*P* = 0.024, Table 2). Mortality was higher in the PJP group (9 cases, 18.0%) than in the non-PJP group (75 cases, 1.6%; Table 2; *P* < 0.001, log-rank test; Fig. 2b). Seven patients died from PJP (Table 2).

### Risk factors for PJP in kidney transplant patients

When we analyzed the risk factors for PJP, donor age at transplantation, desensitization before transplantation, number of HLA mismatches, tacrolimus dose at discharge, acute rejection, and CMV infection were associated with a higher incidence of PJP, and a higher dose of TMP-SMX was associated with a lower incidence of PJP (Table 3). The application of PJP prophylaxis or duration of PJP prophylaxis was not associated with PJP occurrence. In multivariate analysis, donor's age at transplantation (aHR, 1.032; 95% CI, 1.001–1.064; *P* = 0.043), desensitization before transplantation (aHR, 2.261; 95% CI, 1.136–4.498; *P* = 0.020), and tacrolimus dose per body weight at discharge (aHR, 380.747; 95% CI, 7.308–19,835.950; *P* = 0.003) were significant risk factors for PJP (Table 3). However, PJP dose was not independently associated with a lower risk of PJP.

**Table 3:** Risk factors for *Pneumocystis jirovecii* pneumonia after kidney transplantation.

Variables	Univariate			Multivariate		
	HR	95% CI	*P *value	HR	95% CI	*P* value
Recipient age at transplantation	1.018	0.993–1.045	0.159			
Recipient sex (female)	0.963	0.547–1.695	0.895			
Donor age at transplantation	1.031	1.007–1.056	0.011	1.032	1.001–1.064	0.043
Donor sex (female)	1.171	0.673–2.039	0.576			
Cold ischemic time	1.000	1.000–1.000	0.238			
BMI (recipient)	0.891	0.817–0.973	0.010	0.938	0.844–1.043	0.236
Cause of ESRD	1.554	0.889–2.716	0.122			
Delayed graft function	2.053	0.739–5.701	0.168			
Deceased donor	0.700	0.382–1.281	0.247			
Re-transplantation	0.760	0.237–2.442	0.645			
Desensitization	2.133	1.205–3.776	0.009	2.261	1.136–4.498	0.020
Number of HLA mismatch	1.196	0.999–1.430	0.050	1.182	0.947–1.474	0.139
ATG use	0.645	0.290–1.435	0.283			
Tacrolimus dose at discharge (mg)	1.091	1.021–1.165	0.010			
Tacrolimus dose per body weight at discharge (mg/kg)	423.911	11.802–15,226.690	0.001	380.747	7.308–19,835.950	0.003
Tacrolimus level at discharge (ng/mL)	1.026	0.940–1.120	0.562			
Steroid use at discharge	1.004	0.967–1.043	0.825			
MMF use at discharge	0.694	0.250–1.929	0.484			
mTORi use at discharge	3.255	0.790–13.405	0.102			
**Dose of PJP prophylaxis** Double vs. single strength/day	0.230	0.056–0.948	0.042	0.217	0.029–1.603	0.134
Duration of PJP prophylaxis	0.952	0.851–1.065	0.391			

Multivariate analysis was performed using variables that showed significance of *P* < 0.10 in univariate analysis.

*Abbreviations*: ATG, anti-thymocyte globulin; BMI, body mass index; CI, confidence interval; ESRD, end stage renal disease; MMF, mycophenolate mofetil; mTORi, mammalian target of rapamycin inhibitor; PJP, *pneumocystis jirovecii* pneumonia.

### Comparison of PJP according to occurrence time

Among the 50 PJP cases, 25 cases (early PJP) developed within 6 months after transplantation, and 25 cases (late PJP) developed beyond 6 months after transplantation (Table 4). Time from transplantation to PJP occurrence was 3.24 ± 1.48 months in early PJP group and 13.60 ± 7.7 months in late PJP group (*P* < 0.001). When the early and late PJP groups were compared, older females were more likely to have late PJP (Table 4). Time from rejection to PJP onset was longer in late PJP group (7.4 ± 3.8 months) than early PJP group (3.2 ± 0.8 months, *P* = 0.045, Table 4). Duration of prophylaxis before PJP occurrence was 0.04 ± 0.20 months in early PJP and 7.72 ± 7.61 months in late PJP group (*P* < 0.001). The PJP infection during PJP prophylaxis occurred in 24 (96%) patients in the early PJP group and 3 (6%) in the late PJP group. There was no difference in either graft failure (Table 4) or mortality (Table 4; *P* = 0.546, log-rank test, Fig. 2c) between early and late PJP.

**Table 4:** Comparison of *Pneumocystis jirovecii* pneumonia according to occurrence time.

	Early PJP (n = 25)	Late PJP (n = 25)	*P* value
Age at transplantation (years)	47.0 ± 14.5	55.2 ± 11.4	0.055
Sex (female)	6 (24.0%)	14 (56.0%)	0.021
BMI (kg/m^2^)	22.72 ± 0.77	20.90 ± 0.55	0.060
Hemoglobin (g/dL)	11.02 ± 1.60	10.24 ± 2.17	0.154
Delayed graft function	2 (8.0%)	2 (8.0%)	0.434
Re-transplantation, n (%)	2 (8.0%)	1 (4.0%)	0.552
Desensitization, n (%)	11 (44.0%)	8 (32.0%)	0.561
Number of HLA mismatch	3.6 ± 1.4	3.8 ± 1.4	0.554
ATG use, n (%)	2 (8.0%)	5 (20.0%)	0.417
Tacrolimus dose at discharge (mg)	7.67 ± 5.14	6.97 ± 0.99	0.328
Tacrolimus dose per body weight at discharge (mg/kg)	0.12 ± 0.02	0.13 ± 0.02	0.402
Tacrolimus level at discharge (mg/dL)	8.91 ± 0.58	7.18 ± 0.64	0.051
**Formulation of tacrolimus, n (%)**			0.213
Once daily	0 (0.0%)	0 (0.0%)	
Twice daily	21 (84.0%)	16 (64.0%)	
Unknown	4 (16.0%)	9 (36.0%)	
Tacrolimus conversion (twice to once daily), n (%)	1 (4.0%)	0 (0.0%)	0.455
Steroid use at discharge	24 (96.0%)	25 (100.0%)	1.000
MMF use at discharge	23 (92.0%)	23 (92.0%)	1.000
mTORi use at discharge	1 (4.0%)	1 (4.0%)	1.000
PJP Prophylaxis, n (%)	25 (100.0%)	25 (100.0%)	1.000
**Dose of prophylactic TMP-SMP, n (%)**			0.755
1 Single strength/day	24 (96.0%)	24 (96.0%)	
1 Double strength/day	1 (4.0%)	1 (4.0%)	
Duration of PJP prophylaxis (months)	6.1 ± 1.4	5.9 ± 0.6	0.572
Time from transplantation to PJP (months)	3.24 ± 1.48	13.60 ± 7.67	< 0.001
PJP during PJP prophylaxis, n (%)	24 (96.0%)	3 (6.0%)	< 0.001
Graft rejection before PJP, n (%)	5 (20.0%)	8 (32.0%)	0.520
Time from rejection to PJP (months)	3.2 ± 0.8	7.4 ± 3.8	0.045
CMV infection before PJP, n (%)	0 (0.0%)	1 (4.0%)	0.500
Graft loss, n (%)	3 (12.0%)	1 (4.0%)	0.609
Death, n (%)	5 (20.0%)	4 (16.0%)	0.500
Death due to PJP, n (%)	3 (12.0%)	4 (16.0%)	0.500

Data are presented as mean (standard deviation), or n (%).

Abbeviations : ATG, anti-thymocyts globulin; BMI, body mass index; CMV, cytomegalovirus; mTORi, mammalian target of rapamycin inhibitor; PJP, pneumocystis jirovecii pneumonia; TMP-SMX, trimethoprim/sulfamethoxazole.

### Risk factors for early and late PJP in kidney transplant patients

Risk factors for early and late PJP were analyzed using multivariate analysis. The risk for early PJP development was higher in patients with pre-transplant desensitization (aHR, 3.322; 95% CI, 1.239–8.909, *P* = 0.017), higher tacrolimus dose per body weight at discharge (aHR, 315.579; 95% CI, 1.770–56,278.820, *P* = 0.029), or history of rejection (aHR, 69.669; 95% CI, 20.294–239.175, *P* < 0.001, Table 5). In contrast, late PJP was significantly associated with recipient age at transplantation (aHR, 1.052; 95% CI, 1.006–1.101; *P* = 0.026) as well as history of rejection (aHR, 334.679; 95% CI, 95.147–1177.239; *P* < 0.001, Table 5).

**Table 5:** Risk factors for early and late *Pneumocystis jirovecii* pneumonia after kidney transplantation.

**Variables**	Early PJP (n = 50)						Late PJP (n = 50)					
	Univariate			Multivariate			Univariate			Multivariate		
	HR	95% CI	*P* value	HR	95% CI	*P* value	HR	95% CI	*P* value	HR	95% CI	*P* value
Recipient age at transplantation	0.986	0.953–1.019	*0.394*				1.060	1.018–1.104	0.005	1.052	1.006–1.101	0.026
Recipient sex (female)	0.459	0.184–1.150	*0.097*	0.469	0.178–1.238	0.127	1.830	0.831–4.030	0.134			
Donor age at transplantation	1.026	0.993–1.061	*0.122*				1.036	1.002–1.072	0.040	0.995	0.951–1.041	0.831
Donor sex (female)	1.249	0.570–2.738	*0.578*				1.097	0.501–2.405	0.817			
BMI (kg/m^**2**^**)**	0.968	0.864–1.086	*0.584*				0.811	0.710–0.925	0.002	0.884	0.731–1.070	0.207
Delayed graft function	2.062	0.486–8.745	*0.326*				2.026	0.478–8.590	0.338			
Deceased donor	0.417	0.156–1.111	*0.080*	0.895	0.283–2.833	0.850	1.071	0.481–2.384	0.867			
Re-Transplantation	1.057	0.249–4.481	*0.941*				0.485	0.066–3.585	0.478			
Desensitization	2.695	1.223–5.935	*0.014*	3.322	1.239–8.909	0.017	1.656	0.715–3.837	0.239			
Number of HLA mismatch	1.150	0.898–1.472	*0.268*				1.246	0.960–1.617	0.098	1.453	0.947–2.228	0.087
ATG use	0.339	0.080–1.437	*0.133*				1.012	0.380–2.697	0.980			
Tacrolimus dose at discharge	1.101	1.014–1.196	*0.022*				1.073	0.961–1.200	0.212			
Tacrolimus dose per body weight at discharge (mg/kg)	253.346	1.776–36,131.05	*0.029*	315.579	1.770–56,278.820	0.029	775.330	4.495–133,745.5	0.011	115.878	0.140–95,824.920	0.166
Tacrolimus level at discharge (mg/dL)	1.091	0.994–1.198	*0.067*				0.932	0.805–1.080	0.349			
Steroid dose at discharge	1.000	0.949–1.054	*0.996*				1.009	0.957–1.063	0.747			
MMF use at discharge	0.688	0.162–2.916	*0.611*				0.701	0.165–2.974	0.629			
mTORi use at discharge	3.647	0.493–26.960	*0.205*				2.939	0.397–21.761	0.291			
Acute rejection before PJP	93.794	35.169-250.139	< *0.001*	69.669	20.294–239.175	< 0.001	193.108	83.246–447.962	< 0.001	334.679	95.147–1177.239	< 0.001
**Dose of PJP prophylaxis** Double vs. single strength/day	0.237	0.032–1.755	*0.159*				0.225	0.030–1.661	0.143			
Duration of PJP prophylaxis	0.965	0.823–1.131	*0.657*				0.926	0.787–1.091	0.358			

Multivariate analysis was performed using variables that showed significance of *P* < 0.10 in univariate analysis.

*Abbreviations:* ATG, anti-thymocyte globulin; BMI, body mass index; CI, confidence interval; HR, hazard ratio; MMF, mycophenolate mofetil; mTORi, mammalian target of rapamycin inhibitor; PJP, *pneumocystis jirovecii* pneumonia.

## Discussion

This study, showed that late PJP beyond 6 months after kidney transplantation occurred at similar rate as early PJP within the first 6 months after transplantation in the era of short-term universal prophylaxis. When risk factors were analyzed separately for early and late PJP, old age and acute rejection were significant risk factors in late PJP, whereas desensitization, higher tacrolimus dose per body weight at discharge, and acute rejection were associated with early PJP. Although PJP increased mortality, there was no difference in mortality rates between early and late PJP.

The PJP incidence seems to be variable according to risk profiles of kidney transplant populations. PJP incidence ranges from 0.3% in US single-center study to 1.58% in French single-center study^17,18^. German single-center study reported PJP incidence was 3% in ABO-incompatible cases with desensitization whereas PJP did not occur in ABO-compatible cases without desensitization^19^. Proportions of HLA-incompatible and ABO-incompatible kidney transplantation were 7.4% and 1.3%, respectively in US multi-center studies^20,21^. ABO-incompatible kidney transplantation with desensitization in Japan reaches 20–30%, which is as common as that in Korea^22,23^. However, desensitization rate is not high in other countries. Therefore, the PJP incidence (1.033%) in this study would reflect a high proportion of high-risk group with desensitization in this study population (22.3%).

In parallel with previous reports^8–10^, this study demonstrated that heavy immunosuppression, such as desensitization and a higher dose of tacrolimus per body weight at discharge were significant risk factors for PJP. Old donor age was also associated with PJP, in parallel with a previous study that reported expanded-criteria donor including old donor age is a risk factor for PJP^24^. On the other hand, acute rejection rather than old donor age itself might be a true risk factor for PJP considering that group with acute rejection had higher donor age than group without acute rejection (48.99 ± 12.49 vs. 46.75 ± 13.03, *P* < 0.001)^25^. Furthermore, PJP was associated with higher graft failure and mortality than non-PJP. Considering the incidence and significant impact of PJP on clinical outcomes, the survey in this study showed that 85% and 9% of Korean transplantation centers adopted universal and indicated prophylaxis for high-risk groups, respectively. In this study, most patients (n = 4,676, 96.7%) received TMP-SMX prophylaxis.

Universal PJP prophylaxis for 3 to 6 months shifted PJP to the later period^4,18,26,27^. Six months of PJP prophylaxis (6.0 ± 1.0) might have contributed to late PJP in this study. This study showed that late PJP is also harmful for patient survival, similarly to early PJP, as a previous study reported bad impacts of late PJP^27^. Another issue is the high PJP rate (96%) during PJP prophylaxis in the early PJP group. It is uncertain whether this apparent breakthrough infection might have been attributed to resistance to TMP-SMX, underexposure or lack of compliance.

We attempted to elucidate the risk factors for late PJP compared to those for early PJP. A previous episode of acute rejection associated with heavy immunosuppression in the treatment of acute rejection is a significant risk factor for both early and late PJP. Desensitization and tacrolimus dose per body weight at discharge, reflecting initial heavy immunosuppression, were associated with a higher risk for early PJP, but not for late PJP. In contrast, old age was a significant risk factor only for late PJP, suggesting that a patient’s general health condition as well as the degree of immunosuppression are associated with PJP risk in later periods. CMV infection before PJP was reported to be associated with a higher risk of late PJP^28^; however, there was only one CMV infection case among 50 PJP cases and a meaningful analysis could not be performed due to the small case number of CMV infection. Although lymphopenia was suggested as a risk factor for late PJP in previous studies^15,27^, we could not analyze this issue because of the lack of lymphocyte count data in the KOTRY database.

Late PJPs in recent studies are often caused by outbreaks, and centers where outbreaks have occurred are proposed to maintain PJP prophylaxis for more than 12 months^15,29,30^. In Australia, considering cost-effectiveness and high costs of mortality, prophylaxis for 1 year or more is recommended, but this cost-saving must be balanced with adverse events of TMP-SMX, such as increased creatinine levels and the occurrence of resistance ^29,31,32^. Jung et al. reported eight cases of PJP despite 1 year of prophylaxis^33^ and a case of outbreak occurred 10 years after transplantation despite the use of PJP prophylaxis for 1 year^14^, implying that a prolonged duration of prophylaxis may not be the sole solution to late PJP. Setting the proper duration of prophylaxis according to individualized risk assessment could be a better approach, for example longer duration of prophylaxis for old kidney transplant patients with a history of recurrent anti-rejection therapy, reflecting a high risk for late PJP.

The strength of this study is the nationwide analysis of post-kidney transplant PJP based on a nationwide Korean transplant cohort, along with a survey of 32 participating centers regarding their PJP prophylaxis policy. Furthermore, we compared the risk factors and clinical impact of early and late PJP. On the other hand, this study has several limitations. The results in this study with high prevalence of desensitization might not be applicable to other countries with less desensitization. As a registry-based study, for example we could not obtain detailed information, such as complications and lymphocyte counts, as much as those in single center-based studies. Furthermore, we could not analyze the specific cause of apparent breakthrough PJP in early PJP group. A potential discrepancy between drug prescription and actual drug-taking could be another bias in this registry-based study. Further studies with more detailed information are needed to confirm our findings.

In conclusion, late PJP is as common and risky as early PJP and requires individualized risk-based prophylaxis, such as prolonged prophylaxis for old patients with a history of rejection.

## Methods

### Study design and study population

This retrospective cohort study, which used the KOTRY database, a nationwide cohort for solid organ transplantation in Korea, included adult kidney transplant patients who had been registered in the KOTRY from 2014 to 2018, excluding kidney transplant patients who received multi-organ transplantation or were under 18 years old**.** We also conducted a survey to determine the PJP prophylaxis regimens in 32 KOTRY-participating Korean transplantation centers. Standard immunosuppressive protocol consisted of basiliximab induction and triple maintenance therapy using corticosteroid, tacrolimus, and anti-metabolite (mycophenolate mofetil or myfortic acid), which was adopted by 91.4% of patients at discharge. The triple immunosuppression was maintained in 85.9% and 84.6% of patients after 6 months and 1 year after transplantation, respectively. ATG as an induction therapy was mainly used in the high-risk groups, such as patients with preformed donor-specific antibodies.

### Data collection and outcome measurement

Demographic, clinical, and laboratory data were collected at every enrolled center, just before transplantation, at discharge after transplantation, 6 months after transplantation, 1 year after transplantation and at annual intervals. With respect to PJP, information on the occurrence and onset time of PJP was collected with outcome information, such as graft failure and mortality. The presence, dose, and duration of PJP prophylaxis for each patient were also recorded.

### Definitions of clinical outcomes

Positive microbiological study results from bronchoalveolar lavage were considered PJP cases according to the latest international guidelines^34^. CMV infection was defined as a positive whole blood CMV quantitative nucleic acid test result (≥ 34.5 IU/mL)^35^. Graft rejection was defined as biopsy-proven acute T cell-mediated or active antibody-mediated rejection according to Banff criteria^36^. Graft failure was defined as the requirement to return to dialysis or receive re-transplantation. Early and late PJP were defined as PJP that occurred within the first 6 months and 6 months after kidney transplantation, respectively.

### Statistical analysis

Categorical variables are expressed as frequencies or percentages, and continuous variables are expressed as mean ± standard deviation. Data were analyzed using the Chi-squared test, Fisher’s exact test, or Mann–Whitney U test, as appropriate. Graft failure and mortality were estimated using the Kaplan–Meier method and compared using the log-rank test. The risk factors for PJP were analyzed using the Cox proportional hazards analysis. To clarify risk factors for whole, early, and late PJP, multivariate analysis was performed using variables that showed significance (*P* < 0.100) in the univariate analysis. The results are expressed as hazard ratio (HR) and adjusted hazard ratio (aHR), with 95% confidence interval (CI). Statistical significance was set at *P* < 0.050. All data were analyzed using the SAS software (version 9.4; SAS Institute Inc., Cary, NC, USA).

### Ethical approval

The study protocol was approved by the Institutional Review Board of Severance Hospital (approval number: 4–2021-1711). The requirement for informed consent was waived by the Institutional Review Board. This study was performed in accordance with the Declaration of Helsinki 2000 and the Declaration of Istanbul 2008.

## Supplementary Information

Supplementary Information.

## Data Availability

The raw data that support the findings of this study are not publicly available because they were not permitted by KOTRY. However, data can be provided by the authors upon reasonable request with permission from KOTRY.
