# Supplementary material for: Comparison of early and late Pneumocystis jirovecii Pneumonia in kidney transplant patients: the Korean Organ Transplantation Registry (KOTRY) Study
Source: Sci Rep. 2022 Jun 23;12:10682. doi: 10.1038/s41598-022-14580-5 (PMC9226063; doi:10.1038/s41598-022-14580-5)
Supplement: Supplementary file 1 — Supplementary Information. [file 41598_2022_14580_MOESM1_ESM.pdf]

Supplementary Materials for

Comparison of Early and Late *Pneumocystis jirovecii* Pneumonia in Kidney Transplant Patients - the Korean Organ Transplantation RegistY (KOTRY) Study

Gongmyung Lee, M.D., Tai Yeon Koo, M.D., Ph.D., Hyung Woo Kim, M.D., Dong Ryeol Lee, M.D., Ph.D., Dong Won Lee, M.D., Ph.D., Jieun Oh, M.D., Ph.D., Beom Seok Kim, M.D., Ph.D., Myoung Soo Kim, M.D., Ph.D., Jaeseok Yang, M.D., Ph.D.

Supplementary Material File Listing

Supplementary Table S1.

Supplementary Table S2.

Supplementary Table S3.

Supplementary Table S4.

Corresponding author information

Jaeseok Yang, M.D., Ph.D.,

Division of Nephrology, Department of Internal Medicine,

Yonsei University College of Medicine, Severance Hospital

50 Yonsei-ro, Seodaemun-gu, Seoul, 03722, Republic of Korea

Tel.: +82-2-2228-1949, Fax: +82-2-393-6884; E-mail: jcyjs@yuhs.

Supplementary Table S1. Induction therapy

Induction therapy	Non-PJP (n=4,789)	PJP (n=50)	P-value
			0.432
No induction, n (%)	53 (1.1%)	1 (2.0%)	
ATG, n (%)	998 (20.8%)	7 (14.0%)	
Basiliximab, n (%)	3,738 (78.1%)	42 (84.0%)	

Abbreviations: ATG, anti-thymocyte globulin; PJP, *pneumocystis jirovecii* pneumonia.

Supplementary Table S2. Calcineurin inhibitor use at discharge

CNI at discharge	Non-PJP (n=4,789)	PJP (n=50)	P-value
			0.993
No use, n (%)	54 (1.1%)	1 (2.0%)	
Tacrolimus, n (%)	4,584 (95.7%)	47 (94.0%)	
Cyclosporin, n (%)	151 (3.2%)	2 (4.0%)	

Abbreviations: CNI, calcineurin inhibitor; PJP*, pneumocystis jirovecii* pneumonia.

Supplementary Table S3. Comparison of immunosuppressant level among no-PJP, early-PJP group, and late PJP-group

Tacrolimus level (ng/mL)	Early PJP	Late PJP	Non-PJP	P-value
Concentration at discharge	8.91 ± 2.78	7.18 ± 3.08	7.84 ± 3.15	*0.157*
Concentration at 6 months	6.81 ± 2.43	6.70 ± 2.61	6.70 ± 2.54	*0.979*

Note: data are presented as mean ± standard deviation.

Abbreviations: PJP, *pneumocystis jirovecii* pneumonia.

Supplement table S4. Characteristics of donors

Donor	Non-PJP (n=4,789)	PJP (n=50)	P-value
Female, n (%)	2,220 (46.4%)	25 (50.0%)	0.607
Age at transplantation (years)	46.95 ± 13.00	51.32 ± 10.12	*0.004*
Cold ischemic time (min)	140.60 ± 138.30	98.05 ± 97.51	*0.012*
Hypertension, n (%)	721 (15.1%)	7 (14.0%)	*0.876*
Diabetes mellitus, n (%)	248 (5.2%)	0 (0.0%)	*0.179*
eGFR (mL/min/1.73m^2)	84.15 ± 34.08	85.09 ± 27.35	*0.847*

Note: data are presented as mean ± standard deviation, or n (%).

Abbreviations: eGFR, estimated glomerular filtration rate; PJP, pneumocystis jirovecii pneumonia.
